# Obstetrics and Gynæcology

**Published:** 1877-03

**Authors:** 


### III. OBSTETRICS AND GYNÆCOLOGY.

1. *Injections of Hot Water for Arresting Uterine Hæmorrhage.* WINDELBAND. (*Deutsche Med. Wochenschr.* 24, 1876; *Centralbl. f. Chir.*)

Two years ago W. read in some medical journal of two cases of abortion in which Dr. Mann, an Américan physician, had made a successful use of injections of hot water into the vagina and uterus to stop the profuse hæmorrhage. From an extensive experience in a great variety of grave cases of uterine hæmorrhage, the writer has gained the firm conviction that the injections of hot water are an invaluable remedy, far preferable to cold water and the astringents in all cases where prompt assistance is urgently needed.

The injections were always made in the recumbent posture, with a simple fountain syringe. The temperature of the water was 38° (centigrade) in the beginning, to be gradually elevated to 41° C. if necessary. These hot injections never cause any unpleasant or injurious reaction; they are, on the contrary, very grateful to the patient. Their hæmostatic effect seems to be due to their stimulating the contractions of the muscular fibres of the uterus.

2. *Aspirating Movement of the Cervix Uteri.* Galicier. (*La France Médic.*, January 27, No. 8.)

The author established the following phenomena during a speculum examination:

1. The cervix opened by the separation of the two labia, so as to present a funnel-shaped cavity.

2. The labia became more or less approximated or separated according as the speculum was withdrawn or made to penetrate more deeply. No disease of the uterus existed.

The woman was young, and had had one child. She was then in the first month of a second pregnancy.

3. *Imperforate Hymen; Retention of Menses; Death.* Calderin. (*Anal. de Gynecol. Espan.*, May 5, 1876.)

On the 15th of August, a girl, fifteen years old, was seized with acute hypogastric pain. By palpation an abdominal tumor was discovered in that region, and vesical catheterism was practiced to facilitate the diagnosis. The escape of a large quantity of urine procured manifest relief. This lasted until January 19th, when the same symptoms were presented, with greater intensity. The same treatment was then adopted, without effect, when a consultation was held, and it was discovered that a soft fluctuating tumor filled the pelvic cavity and extended to the umbilicus, the hymen being imperforate. Menstrual retention was diagnosticated, and puncture proposed.

The operation was performed on the 23d with a trocar, with the effect of giving exit to a chamber full of blood. All went well till the 28th, when there was right hypochondriac pain, fever with a pallid countenance, and the escape of a large quantity of fetid pus from the vagina. On the following days the pain extended to all parts of the abdomen. Cadaveric aspect, bilious vomiting, precordial anxiety, small pulse, hiccough, and retention of urine. Catheterism, morphia, belladonna and cataplasms gave small relief. This general condition — the patient being sometimes better and sometimes worse — lasted until the 6th of February, the patient dying on the 7th.

Considering the age of the patient, the volume of the tumor, and the antecedents of the family (the menses appeared at the same age with her sisters), the physicians concluded that the retention had dated one year before, and that the tumor was formed exclusively by vaginal dilatation. An autopsy was not permitted. The hepatic symptoms could be referred (according to Hervieux) only to peritonitis or phlebitis. Now in this case there was no meteorism — a most constant symptom in peritonitis — and the death therefore must have resulted from phlebitis — the result of exten_ sive prolonged congestion of the vessels of the uterus and vagina. To this is to be added the sudden admission of air to the vagina, the general depressed condition of the system, and the inflammatory manifestations which preceded the very inoffensive operation.

Bernutz (*Monographie sur les rétentions menstruelles*) corroborates this opinion, and proposes in such cases to withdraw the blood at several sittings with a capillary trocar, and to incise the hymen only when previous evacuations have permitted the organs distended by the retained blood to return to their primitive state.

4. *Treatment of Premature Rupture of the Bag of Waters.* Di Pineiro. (*Anal. de la Soc. gyn. Espan.*, May, 1876.)

When this accident occurs at the fundus of the uterus, it is relatively unimportant; but when it is produced at the neck, death of the fœtus may ensue, or serious maternal trouble. Different measures have been proposed by various authors for the management of dry labors (bleeding, general and local baths, unguents, belladonna to the cervix, etc.), but all are of doubtful utility. The author proposes the following:

In such cases what actually occurs? The uterus, accustomed to contract upon a smooth and uniformly equal surface, enfolds a body of irregular surface, such as the fœtus; the contractions are no longer uniform, and the progress of dilatation of the os is prolonged. The different points of the fœtus support unequal degrees of pressure, whence a loss of equilibrium results in the circulation, and the parts not pressed upon become congested, e. g., the encephalon. On the other hand, the bag of waters operates as a wedge in the process of dilatation; but when it is prematurely emptied there is a new cause for retardation of the expansion of the os, and, besides, the head is unduly compressed, so that cephalhæmatoma may occur. For the mother, then, the labor is prolonged, and the danger proportionately increased; for the fœtus, there is an added probability of cephalhæmatoma, congestion and death.

The author, taking nature for his guide, proposes to employ hydrostatic dilatation. By this agent the os is enlarged without fear of contusion or tearing, and the pressure is not only made uniform, but can also be regulated. The presenting part of the fœtus cannot possibly be compressed. The uterine orifice is closed with an obturating dilator, so that the issue of such amniotic fluid as has not been evacuated at the instant of rupture is prevented, and thus the danger of congestion is set aside. In short, the prospect is much more favorable for the termination of the labor, in a manner satisfactory to both the mother and the child.

It is clear that one dilator is not sufficient; in fact, that various sizes will be requisite. The author has devised dilators constructed of caoutchouc, in the form of a figure eight, with concave extremities, in order to be adapted to the presenting part of the fœtus. Their introduction is readily effected. It is only necessary to take a gum elastic sound, insinuate it into a little pocket purposely made in the dilator, roll the latter about the sound, and insert it into the uterine orifice, making use of the right index finger as a guide. This done, the sound is retracted, and the dilator is held *in situ* with the finger until its gradual distension by the injection of water. Once in place, there is no fear of its slipping, as it is held by its projecting extremities. No. 1 is successively replaced by Nos. 2 and 3, until dilatation is complete, and the delivery then is permitted to progress in the natural way.

In certain presentations, of course, the introduction of the dilator is more difficult than in others; but this difficulty is proportionately less according as the rupture is the more recent.

In tetanic contractions, inhalations of the nitrite of amyl may be employed with benefit.

